# Scaling of the Parameters for Cost Balancing in Self-Organized Task Switching

**DOI:** 10.5334/joc.137

**Published:** 2021-01-18

**Authors:** Irina Monno, Markus Spitzer, Jeff Miller, David Dignath, Andrea Kiesel

**Affiliations:** 1 University of Freiburg, DE; 2 University of Otago, NZ

**Keywords:** multitasking, voluntary task-switching, switch costs

## Abstract

Previous studies on voluntary task switching using the self-organized task switching paradigm suggest that task performance and task selection in multitasking are related. When deciding between two tasks, the stimulus associated with a task repetition occurred with a stimulus onset asynchrony (*SOA*) that continuously increased with the number of repetitions, while the stimulus associated with a task switch was immediately available. Thus, the waiting time for the repetition stimulus increased with number of consecutive task repetitions. Two main results were shown: first, switch costs and voluntary switch rates correlated negatively – the smaller the switch costs, the larger the switch rates. Second, participants switched tasks when switch costs and waiting time for the repetition stimulus were similar. In the present study, we varied the SOA that increased with number of task repetitions (*SOA increment*) and also varied the size of the switch costs by varying the intertrial interval. We examined which combination of SOA increment and switch costs maximizes participants’ attempts to balance waiting time and switch costs in self-organized task switching. We found that small SOA increments allow for fine-grained adaptation and that participants can best balance their switch costs and waiting times in settings with medium switch costs and small SOA increments. In addition, correlational analyses indicate relations between individual switch costs and individual switch rates across participants.

In our daily life we often engage in multitasking. Yet, our ability to multitask is rather limited. Empirical research has highlighted these limitations, for instance, by demonstrating that switching between two or more tasks is associated with performance costs (for reviews see e.g. Kiesel, et al., 2010; Koch, et al., 2018; Monsell, 2003; Vandierendonck, et al., 2010). While research on switch costs captures a unique aspect of everyday multitasking, it neglects important factors other than performance. For example, when multitasking, it is often not only critical *how* to perform multiple tasks in rapid succession, but also relevant to select *what* task to perform in the first place (Arrington & Weaver, 2015; Braun & Arrington, 2018; Kiesel & Dignath, 2017; Dignath, et al., 2015; Schuch & Dignath, 2020). Indeed, a rich research tradition on decision making provides evidence that people consider different costs and benefits for their decisions (i.e., Basten et al., 2010; Kool et al., 2010; Simen et al., 2009) and recent theoretical work suggests that such utility-based decisions also take into account performance costs in multitasking (Shenhav et al., 2013, Musslick et al., 2015; Shenhav et al., 2016; see also Schuch, et al., 2019). Against this background, the present study examined the structure of such cost/benefit arbitrations in more detail. Going beyond previous research, we asked how different determinants of performance during task switching inform task selection.

The link between task performance and task selection in voluntary task switching was proposed by Arrington and Logan (2004; 2005). In some studies participants could freely choose between a magnitude or parity judgement task, and were instructed to perform each task on about half of the trials as well as in a random order (Arrington & Logan, 2004; 2005); we refer to this as the randomness instruction in the following. Regarding task performance, the voluntary task switching (VTS) resulted in switch costs (differences in response times, RTs, and error rates between repetition trials and switch trials) suggesting that cognitive processes associated with task switching are time-consuming (Arrington, et al., 2014). Moreover, task selection behavior was biased in favor of a higher repetition rate. Participants repeated tasks more often than expected by chance (about 35% switch rate) violating the instruction to select tasks randomly (e.g., Arrington & Logan, 2005; Yeung, 2010).

Importantly, in voluntary task switching experimental factors determining the size of switch costs also influenced the proportion of task switches, or put differently, the repetition bias (Arrington & Logan, 2004; 2005). More specifically, both the tendency to repeat the task and the switch costs decreased with a longer duration of the intertrial interval (ITI). The reduction of switch costs as a consequence of prolonged ITI could be explained by actively controlled preparation for the next task, which can be partly finished before the new stimulus is displayed (Arrington & Logan, 2004; Arrington 2008; Liefooghe et al., 2009). Alternatively, Mayer and Bell (2006; see also Vandierendonck et al., 2010, for an overview) explained the ITI effect by assuming that passive activation of the task set decayed over time after the response was executed. However, the less time-costly task switching is, e.g. after long ITIs, the more often task switches are chosen, that is the repetition bias decreases.

Further observations showed that task selection can be influenced by varying the occurrence of stimuli. For example, stimulus repetition was negatively correlated with the number of voluntary task switches (Arrington, & Logan, 2005, Exp. 4 and 5; Mayr, & Bell, 2006). Moreover, Arrington (2008; see also Arrington & Weaver, 2015) showed that the voluntary task choice was influenced by stimulus availability. In two experiments participants had to decide between a letter (vowel/consonant) and a number (odd/even) judgment task. Stimulus availability was manipulated by presenting the two stimuli in each trial with a variable SOA (0, 50, 100, 150 ms). Additionally, Experiment 2 included the manipulation of ITI (400 and 2000 ms). The task associated with the first stimulus was selected more frequently in accordance with the increasing SOA, which supports the impact of stimulus availability on voluntary task choice. However, Experiment 2 showed that when the ITI was larger, the effect of SOA was weaker, providing further evidence that the ITI duration affects task selection and also interacts with the availability of the target stimulus (Arrington, 2008).

Assuming a link between task selection and performance costs, Reissland and Manzey, (2016; see also Brüning, & Manzey, 2018) proposed that the repetition bias might indicate strategic adaptation of our cognitive systems. Possibly people try to avoid the effort associated with switching tasks and thus prefer to repeat the task when they are free to choose which task to perform (Kiesel & Dignath, 2017; Mittelstädt, et al., 2018a). In line with this assumption, in studies without the randomness instruction, in which participants were instructed to freely choose the task in each trial, the switch rate was low (Kessler et al., 2009; Arrington & Reiman, 2015).

Support for the idea of strategic task selection is also provided by studies of voluntary task switching with reward. That is, when voluntary task switches (Braun & Arrington, 2018; Braem, 2017; Fröber & Dreisbach, 2017) are associated with reward, participants decided to switch tasks more often. Similarly, when participants are signaled that the reward for successful performance will increase in the next trial (i.e., increased reward prospect), voluntary switching rates increased (Fröber & Dreisbach, 2016; Fröber, et al., 2018, Jurczyk, et al., 2019) even if the switch-related task was more difficult than the previously performed task (Jurczyk, et al., 2019). Interestingly, it has been shown that the voluntary switching rate decreases if the reward prospects remain high. These results are in line with studies which examined the impact of cost-benefit balancing on decision making (Basten et al., 2010).

Since Arrington and Logan (2005) have concluded that voluntary task switching can involve both strategic and stimulus-driven control processes, the voluntary task switching paradigm seems to be a suitable tool to study the interaction between different sources of cognitive control as well as the interdependence of task performance and task selection. Yet, the randomness instruction remains a critical point, because following this instruction requires additional control processes to generate random task sequences (Baddeley, 1966). As a consequence, the observed voluntary switch costs may also reflect further costs related to additional control processes involved in the generation of these random task sequences.

Additionally, the observed repetition bias can be considered as a violation of the randomness instruction. Thus, the repetition (or switch) rate in the original voluntary task switching paradigm is only an indirect indicator of the voluntary task selection. In the present study we are especially interested in the interplay of task performance and task selection as well as in the factors determining the decision to switch the task. The novel variant of the voluntary task switching paradigm allows us to investigate the different factors influencing performance costs and task selection in multitasking without the randomness instruction. In this study, our specific aim was to explore which parameters of this novel task switching variant enable participant to most effectively select tasks based on their performance costs.

## SELF-ORGANIZED TASK SWITCHING

**Figure 1:**
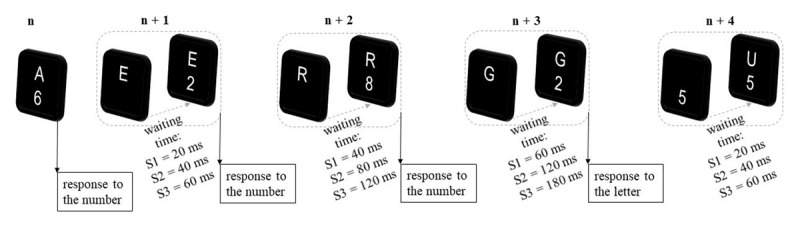
Exemplary Illustration of Accumulation of SOA increments Used in Specific Session (S1, S2, S3) to switch SOA. *Note: n* = first trial in block. After the response to the number task, the waiting time for the number in the first repetition trial (*n* + 1) is equivalent to the SOA increment. In subsequent trials waiting time prolongs according to the specific SOA increment. The observed waiting time in the switch trial (*n* + 3) is recorded as the switch SOA. In the trial after the task switch (*n* + 4) the waiting time starts anew.

In the self-organized task switching paradigm (Mittelstädt et al., 2018b; 2019) participants were instructed to optimize their overall task performance, i.e. to execute each experimental block as quickly and accurately as possible. To induce task switches, stimulus presentation was delayed for the task repetition stimulus relative to the task switch stimulus. More specifically, two stimuli (a letter and a digit) related to two different tasks (vowel/consonant or odd/even) were presented in each trial. After performing one of the two tasks, the stimulus associated with the task repetition was displayed in the next trial with a delay (SOA). In contrast, the switch-related alternative stimulus was immediately available. Importantly, this delay increased with each consecutive task repetition by a fixed SOA increment until one decided to switch tasks. We refer to the delay between the switch and repetition stimulus as waiting time. In addition, in the trial in which the participant switched to the other task we refer to the waiting time of this trial as the switch SOA. This time indicates the waiting time for the repetition stimulus at which the participant decided to switch (see ***Figure 1***).

Similar to the study by Arrington and Logan (2004, 2005) participants performed both tasks equally often, but without being explicitly asked to do so. Rather, the number of trials per task in each block was externally determined so that both tasks were completed the same number of times. This means that once a participant had finished the trials for one task in a block, only the stimulus for the remaining task was presented in the remaining trials of this block. Importantly, the data analyses did not consider these trials because participants were not able to select tasks.

In this setting, with each consecutive task repetition participants have to consider increased waiting times for the repetition stimulus, while a task switch is always compensated with the reset of the waiting time. Consequently, task selection in favor of switching is potentially facilitated by the fact that the immediate onset of the switch-related stimulus can trigger the corresponding task set and at the same time the activation of the repetition-related task set decreases while waiting for its stimulus. Thus, the likelihood of switching tasks should be increased because the switch-associated task set passes the task activation-selection threshold first and thus wins against the repetition task set. In line with these predictions, results showed that two factors impacted on switch rates. For larger SOA increments (for which the waiting times for the repetition stimuli increased faster with consecutive task repetitions), switch rates increased. Also, for longer ITIs (for which task switch costs are reduced), switch rates increased. Moreover, participants avoided waiting too long for the repetition stimulus and switched tasks especially when the difference between switch SOA and switch costs was small. In sum, the results from Mittelstädt et al. (2018b; 2019) revealed further evidence for the link between task performance and task selection and support a trade-off between different types of costs during voluntary task selection.

Importantly, the self-organized task switching paradigm allows researchers to measure both task performance (i.e. switch costs) and task selection behavior (i.e. switch SOA) on a common time scale. As Mittelstädt et al. (2018b, 2019) previously showed, in conditions with short ITIs, participants were quite good at adapting their switch SOA according to their switch costs. However, although the difference was not significant, the switch SOA was about 100 ms longer than the switch costs and there was quite some inter-individual variance. Interestingly, Reissland and Manzey (2016; see also Brüning, & Manzey, 2018) also provided evidence for inter-individual differences in dealing with multitasking situations. More recently, Brüning et al. (2020) demonstrated that individual differences in task processing mode correspond to individual differences in response organization.

The present study aimed to extend the existing findings and investigate exactly how the interplay of switch costs and SOA increment influences the decision to switch tasks. Specifically, we manipulated switch costs by varying the ITI in three steps and the SOA increment also in three steps and expected to identify the conditions under which participants are able to match their switch costs and switch SOA most precisely, i.e. to trade-off most efficiently between switch costs and waiting times for the repetition stimulus. In addition, we aimed to gain further insight into whether an individuals’ ability to balance their time costs is related to their task selection behavior.

## THE PRESENT EXPERIMENT

In the present research we investigated how the relative scaling of SOA increment and switch costs impacts on the cost-benefit arbitration that guides task selection. In the self-organized task switching paradigm, the ratio of SOA increment and switch costs must be crucial for participants to trade-off waiting time and switch cost for methodological reasons. If the SOA increment is large (e.g. 60 ms) while switch costs are small (e.g. 100 ms), no fine-tuning of waiting time and switch cost is possible because in this example participants can only switch at either 60 or 120 ms waiting time. Yet, if the SOA increment is small (e.g. 20 ms) while switch costs are large (e.g. 300 ms), participants would have to switch to the alternative task after a waiting time of 300 ms. This waiting time would only be reached after 15 repetition trials because of the small SOA increment. In this example it might be that participants would hardly switch at all because the setting promotes frequent task repetitions.

To manipulate waiting time, three different SOA increments were used (20 ms, 40 ms, 60 ms; see ***Figure 1***). To manipulate switch costs, three different ITIs were used (0 ms, 250 ms, and 700 ms) that have been shown to modulate the size of switch costs effectively and resulted in large (ca. 300 ms), medium (ca. 200 ms) or small (ca. 100 ms) switch costs (Arrington, 2008; Mittelstädt et al., 2019). This full factorial design allowed us to determine which ITI/SOA increment combination allows an optimal tradeoff.

## METHOD

### EXPERIMENTAL DESIGN

The present study was conducted using a within-subject design. Each participant was tested in three sessions that differed in the use of one of the three SOA increments. Within a session we implemented three different ITIs which varied blockwise. Both the order of the sessions and the order of the blocks was counterbalanced across participants. Our manipulation resulted in nine varying ITI/SOA increment combinations: 0/+20, 0/+40, 0/+60, 250/+20, 250/+40, 250/+60, 700/+20, 700/+40, 700/+60.

### PARTICIPANTS

118 students from the University of Freiburg or residents of Freiburg (35 males; 15 left-handed; *M*_age_ = 24.3 [*SD* = 5.2]) participated in the experiment and received either course credit or financial remuneration. All participants had normal or corrected to normal vision and were treated according to the ethical standards of the American Psychological Association. The sample size was based on a-priori power analyses (*α* = 05, *1–β* = .95) with G*Power 3.1 (Faul, Erdfelder, Buchner, & Lang, 2009); these indicated that 107 participants would be needed to detect differences of .5 between two z-transformed Pearson *r’s*. For reasons of counterbalancing and with respect to the possibility that participants could be excluded due to a lack of task switch, we increased the number of participants to *N* = 118.

### APPARATUS AND STIMULI

The experiment was run with E-Prime software on a Fujitsu Eprimo P920 computer with a 24 inch monitor. Participants were placed in front of a computer screen at a viewing distance of approximately 60 cm. They responded with the index and middle fingers using the “y”, “x”, “,” and “.” keys on a QWERTZ keyboard, which was positioned centrally on the table. The fingers of each hand were mapped to one task and the specific S-R mappings were counterbalanced across participants.

The stimuli of the two tasks were presented one above the other within a white fixation rectangle (11 mm × 19 mm) in the center of the black screen. Target stimuli were white colored numbers 2–9 for the number task (odd/even) and capital letters A, E, G, I, K, M, R, and U for the letter task (consonant/vowel), measuring approximately 5 × 7 mm. Neither the same numbers nor the same letters appeared in successive trials. The position of the stimuli remained the same in the block, but changed over the blocks. In the first block, the number stimulus was presented at the top and the letter stimulus at the bottom for the half of participants. The other half of participants began with the block in which the stimuli-location mapping was reversed. There were also training blocks with instructed task, and in these a white arrow was presented outside the rectangle at the corresponding position (e.g. top/bottom) to indicate the task that should be performed in the current trial.

### PROCEDURE

**Figure 2:**
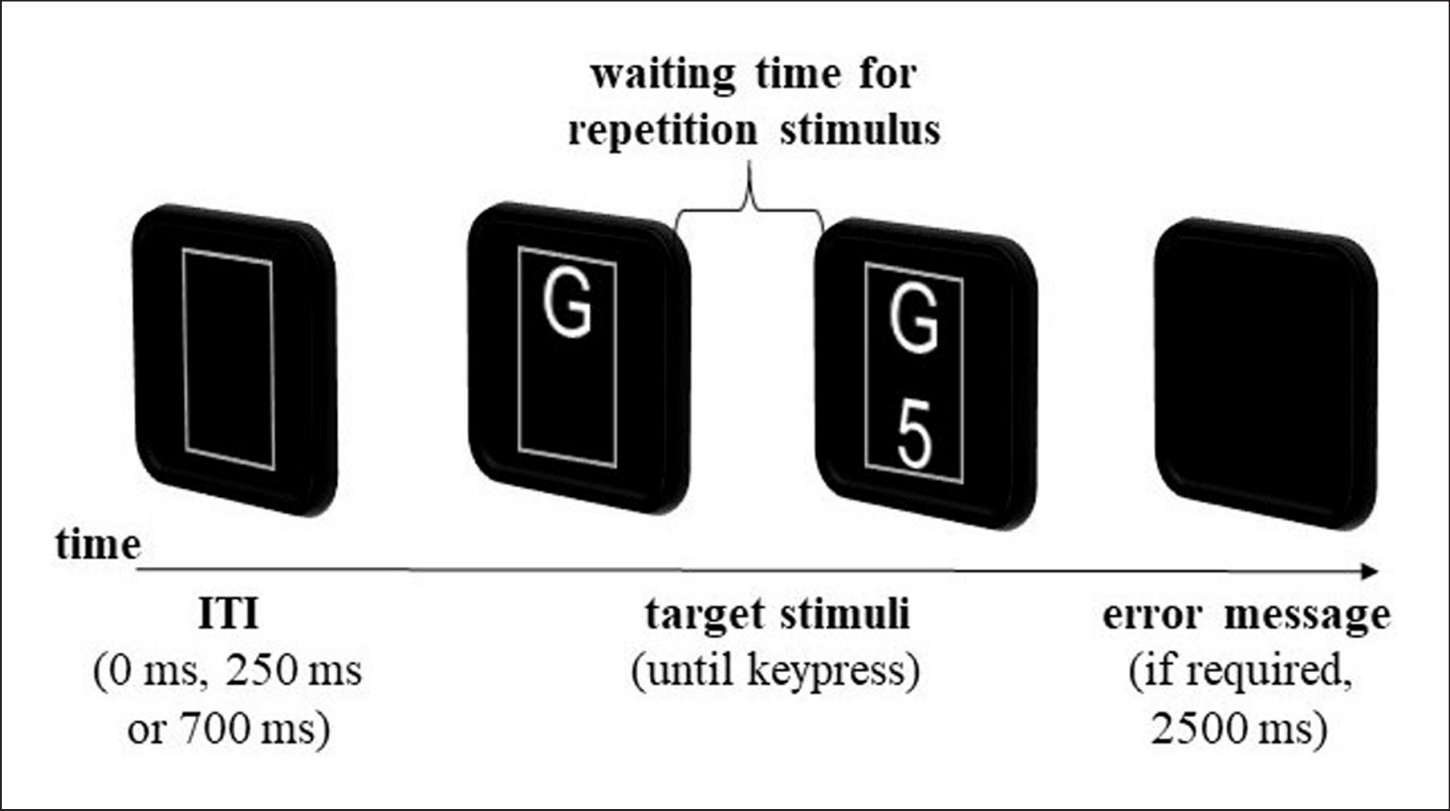
Typical Trial Structure in the Free-Choice Blocks. *Note*: Waiting time for repetition stimulus is equivalent to the number of task repetitions × specific SOA increment (20 ms, 40 ms or 60 ms). SOA increments varied between the sessions, ITI duration varied blockwise.

The procedure of all sessions was the same except for the SOA increment used, which varied between 20, 40, and 60 ms across the sessions. The order of these sessions was counterbalanced across participants. In each session, we applied 15 blocks (3 training blocks, 12 test blocks) with three variable ITIs (0 ms, 250 ms, 700 ms; see ***Figure 2***). The ITIs were constant within a block, but varied across the blocks. Therefore, in each session participants were first trained in three blocks of 60 trials per block (30 number tasks and 30 letter tasks, 180 trials in total) in three different ITI conditions, performing the letter and the number tasks in an alternating-runs procedure (forced-choice blocks; see Rogers & Monsell, 1995). In addition, the implementation of forced-choice blocks ensured that the participants were aware of the possibility to switch tasks during the block. Subsequently, in twelve test blocks of 90 trials (45 number tasks and 45 letter tasks, 1080 trials in total) participants decided by themselves which task to complete in each trial (free-choice blocks). In each block, one of the ITIs was used, so there were four free-choice blocks with the same ITI in each session. The order of the blocks was counterbalanced between the participants and arranged so that the ITI never repeated in two successive blocks.

As shown in ***Figure 2***, at the beginning of each trial, a black display with a gray fixation rectangle appeared for the duration of the current ITI. Then the color of the rectangle changed to white, and the stimuli were presented inside of the rectangle. In the first trial of a block, the two stimuli were displayed at the same time. In the following trials, only the task-switch stimulus appeared directly at the beginning of a trial, whereas the task-repetition stimulus was delayed by the amount of the current accumulated SOA increment. In other words, each task repetition was accompanied by the waiting time for the repetition stimulus. Importantly, the waiting time increased depending on the number of repetitions. The collected RTs were the time duration from onset of the stimulus that appeared first until participant pressed the response key. Hence, to get the true RTs for repetition trials, we subtracted the current SOA from the total time. For the switch trials RTs this calculation was not necessary, because the stimulus for the switch task was presented without the delay and the collected RTs were the true switch RTs. After the fixed total number of the same task type was completed within a block, a placeholder (i.e., “#”-sign) occurred at the corresponding position in the rectangle and participants had to perform the other task for the remainder of the trials in the block. In the forced-choice blocks both tasks/stimuli always appeared simultaneously. At the same time, the white arrow was displayed beside the rectangle at the corresponding position to indicate which task had to be performed in the current trial. The participants were instructed to complete the tasks as quickly as possible and in free-choice blocks, participants were additionally instructed that they could decide themselves which task to perform in each trial with the goal to be as fast as possible and to avoid errors. Similar to the study from Mittelstädt et al. (2019), participants received the following instructions in German:

“You have to perform 90 tasks in one block (= 90 trials). You can decide which task to perform in a trial, as long as both tasks are available. Select the tasks to be as fast as possible without committing errors. Reaction time measurement in each trial starts with the presentation of the first task (or of the “#”–sign) and the change of the rectangle color to white.”

In case of error, a short version of the instructions with the task and response-key mappings was presented for 2500 ms. After each block, participants received performance feedback (i.e., mean collected response time and number of errors) and could take self-paced breaks.

### DATA ANALYSIS OVERVIEW

To identify the optimal ITI/SOA increment combination we choose the following data analysis procedure. First, we examined aggregate measures of task performance and task selection for each ITI/SOA increment combination. As an indicator of task performance, we calculated means of the median switch cost for each ITI/SOA increment combination (see Mittelstädt, et al., 2019, for the rationale of why medians instead of means over individual data points were assessed). As an indicator of task selection we used two measures, (i) mean switch rate and (ii) mean of the median switch SOA (median SOA at which participant decided to switch) for each condition. Second, we calculated correlations between switch costs and switch rates and between switch costs and switch SOAs separately for each ITI/SOA combination to examine individual differences in each condition.

Third, to identify which ITI/SOA increment combination allows for an optimal tradeoff of costs, we examined the relationship between task performance and task selection on both the averaged and individual levels. Since both switch costs (task performance) and switch SOA (task selection), refer to the same scale (i.e., time measured in milliseconds), we calculated the individual difference score (Diff Score = median switch SOA – median RT switch cost) for each participant in each ITI/SOA increment combination. To examine whether the difference between switch costs and switch SOA was significant, we carried out the t-tests against zero for the averaged Diff Scores of each condition. The condition with the smallest and non-significant averaged difference should allow the most efficient trade-off between switch costs and switch SOA. Using the open source statistical program JASP Version 0.11.1 (JASP Team, 2019), we also computed the Bayes factor BF_01_ (default Cauchy prior width = .707), which quantifies the evidence for the absence of the difference (null hypothesis) against the alternative hypothesis. To compare the trade-off points of the different conditions, we ran a 3 × 3 repeated measurements ANOVA with the factors ITI and SOA increment, and the dependent variable Diff Score. As a second indicator of the trade-off ability, we considered the relation of the switch costs and switch rate on the individual level. We reasoned that if participants could more efficiently balance switch costs and switch SOA in a condition, the correlation between switch costs and switch rates in this condition would be stronger than in a condition with a less successful trade-off. Therefore, we compared the magnitude of the correlation between switch costs and switch rates for each ITI/SOA increment combination.

Finally, in order to investigate more directly how an individuals’ trade-off ability is related to their task selection behavior, we performed a correlation analysis between switch rate and the absolute value of the difference score.^1^ Note, with this analysis we aimed to examine whether participants who can better balance switch SOA and switch costs, indicated by a small individual difference score, also switched tasks more often than the participants with lower trade-off ability.

## RESULTS

### DATA PREPARATION

Since the forced-choice blocks were part of the task training and our focus of interest was to investigate the relationship between voluntary task selection and task performance, we analyzed the participant’s RTs only in the free choice blocks. Similar to Mittelstädt et al. (2018) we checked whether the switch rate in the first free-choice block was significantly smaller than in subsequent blocks. As shown in Table A.1 in the Appendix, this was not dramatically the case; therefore, we did not exclude any free-choice blocks from the analyses.

As in previous self-organized task-switching studies (Mittelstädt et al., 2018; 2019) we excluded the following trials from the analysis: the first trial of each block, error trials (5.00 %) and trials following an error, trials in which participants pressed the key before stimulus onset (.40%), any trials without the possibility to choose between the two tasks (i.e., any trials when a placeholder was presented for one task, 14.00 %), and trials with RTs less than 200 ms (.10 %) or greater than 3000 ms (.02 %).

### PARTICIPANT EXCLUSION PROCEDURE

First, we excluded one participant with an error rate of 30.00 %. In the next step, we examined whether participants showed extreme task choice behavior. We identified 7 participants who only switched (99.96 % task switches) as well as 8 participants who only repeated the tasks (100.00 % task repetitions) in all nine conditions and removed their data from the analysis. Then, we ensured that after the data preparation each participant had a substantial number of repetition and switch trials in each condition. We applied the cut-off of a minimum of 10 trials to get a meaningful estimation of individual switch costs. If the minimum number had not been fulfilled, the respective participants were excluded from the analysis only in the particular condition (see row “*N*” in ***Table 1*** for the remaining sample size in each condition).

**Table 1:** Mean Median Switch Costs, Switch Rates, Mean Median Switch SOA, Separately for Each ITI/SOA increment Combination.


	ITI 0 MS			ITI 250 MS			ITI 700 MS		

	SOA +20 MS	SOA +40 MS	SOA +60 MS	SOA +20 MS	SOA +40 MS	SOA +60 MS	SOA +20 MS	SOA +40 MS	SOA +60 MS

Switch costs (ms)	154 (137)	141 (111)	158 (104)	78 (90)	75 (90)	68 (82)	38 (67)	38 (60)	55 (112)

Switch rate (%)	25 (22)	30 (23)	31 (19)	30 (22)	38 (22)	44 (24)	38 (23)	43 (23)	49 (23)

Switch SOA (ms)	114 (85)	182 (135)	235 (147)	88 (75)	126 (106)	163 (120)	63 (46)	108 (85)	141 (113)

N	82	91	93	83	87	93	86	89	90


*Note*: Switch costs = task switch RT – task repetition RT, Standard errors of the means in parentheses. *N* = remaining sample size in each condition after participants exclusion procedure.

### TASK PERFORMANCE

First, we examined whether the size of switch costs varied according to the duration of the ITI. Similar to Mittelstädt et al. (2019), we calculated the median switch costs (median RT switch – median RT repetition) for each participant in each ITI/SOA increment combination. ***Table 1*** provides an overview of the average switch cost for each condition. As expected, participants had the highest switch costs in blocks with shortest ITI of 0 ms and lowest switch costs in blocks with longest ITI of 700 ms. Importantly, within a given ITI, the switch costs did not seem to differ substantially between the different SOA increments.

### TASK CHOICE BEHAVIOR

Next, we analyzed participants’ task choice behavior in each condition. In detail, we examined how often participants decided to switch to the alternative task in each condition (i.e. the switch rate for each condition) and at which switch SOA they decided to switch. Thus, we calculated each participant’s individual switch rate and individual median switch SOA per condition. Note, according to the SOA increments used in this experiment, the corresponding median switch SOA varied in discrete steps (Mittelstädt et al., 2019). Therefore, similar to Mittelstädt et al. (2019) we used interpolated median switch SOA for each participant in each condition to get more fine-grained estimates.

As shown in ***Table 1*** the average switch rate was the lowest in the ITI 0/SOA +20 condition (25 % switches) and highest in the ITI 700/SOA +60 condition (49% switches). Moreover, we observed that increases in ITI and SOA increments led to higher switch rates. A two-way repeated measures ANOVA was performed to evaluate the effects of ITI and SOA increment on participants’ switch rates. There were statistically significant main effects of ITI and SOA increment, *F(2,130)* = 88.41, *p* < .001, *η_p_^2^* = .58, and *F(2,130)* = 18.73, *p* < .001, *η_p_^2^* = .22, respectively. The two-way interaction failed to reach significance, *F(4,260)* = .72, *p* = .578, *η_p_^2^* = .01.

The average switch SOA varied across different conditions from 63 ms with ITI 700/SOA +20 to 235 ms with ITI 0/SOA +60 (see ***Table 1***).

### INDIVIDUAL DIFFERENCES IN THE RELATIONSHIP BETWEEN TASK PERFORMANCE AND TASK CHOICE BEHAVIOR

**Figure 3:**
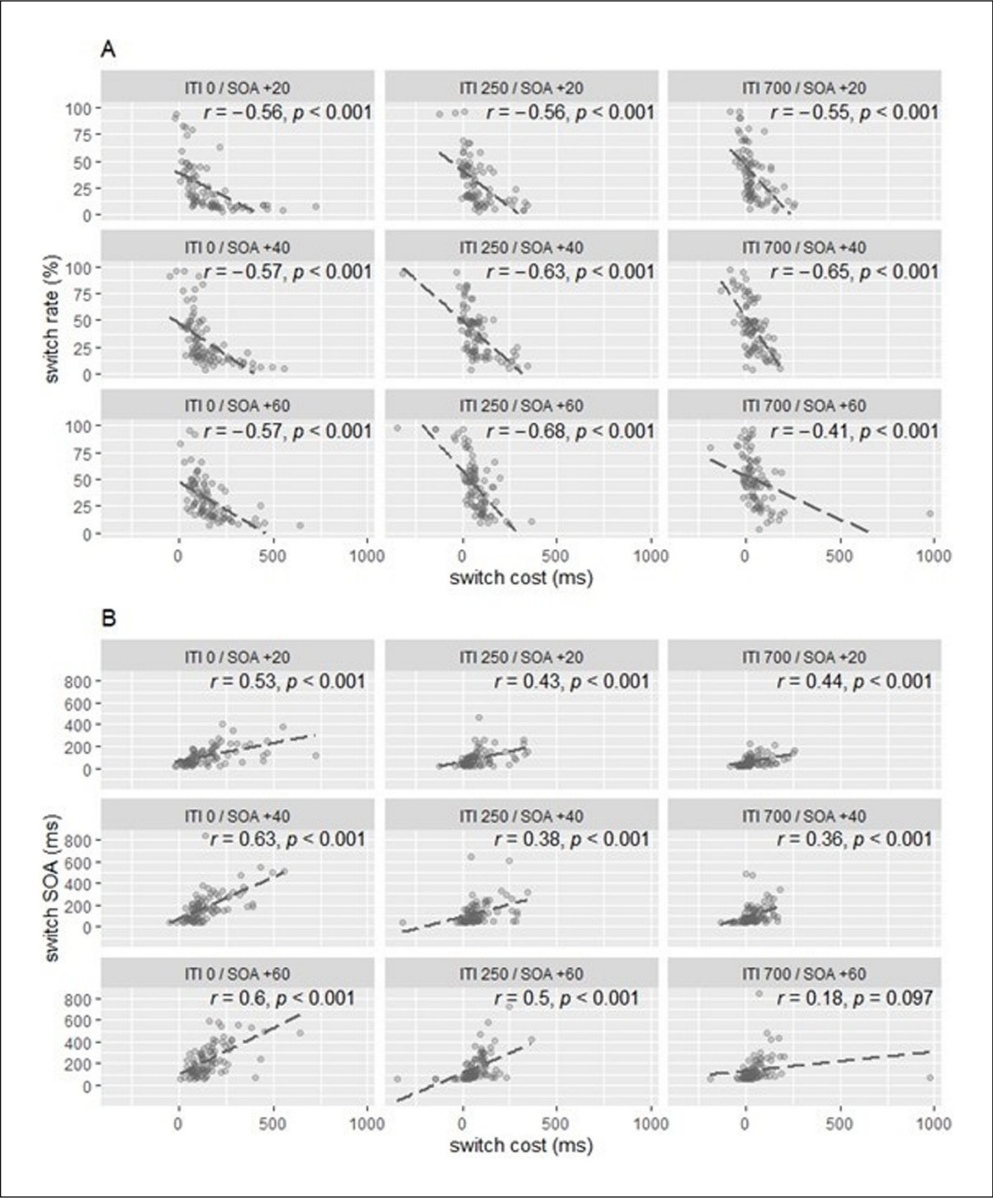
Relationship between Task Performance and Task Selection in each ITI/SOA increment Combination. *Note*: Panel **A** displays scatterplots of individual median switch costs against individual switch rates. Panel **B** displays scatterplots of individual median switch costs against the individual median SOAs of switch trials. Dashed lines represent the corresponding regression lines.

Pearson’s correlation coefficients were computed across participants for each ITI/SOA increment combination to assess the relationship between switch costs and switch rates and the relationship between switch costs and switch SOA. As shown in ***Figure 3*** there were strong negative correlations between switch costs and switch rates, with the strongest correlation being observed in the ITI 250/SOA +60 combination, *r* = –.68 (***Figure 3A***). Switch SOA and switch costs correlated positively across all ITI/SOA increment combinations; the correlation coefficients varied between *r* = .18 and *r* = .63 (***Figure 3B***).

With one exception apparently resulting from an aberrant data point, all correlation coefficients were significant, *p* < .001.^2^

### TRADE-OFF BETWEEN SWITCH COSTS AND SWITCH SOA

As is evident in ***Table 2***, the smallest mean Diff Score was observed in ITI 250/SOA +20 condition indicating the smallest difference between switch costs and switch SOA. A t-test against “0” showed that the difference between switch costs and switch SOA was not significant for the ITI 250/SOA +20 combination (*p* = .303). An additional Bayesian t-test revealed moderate evidence for the absence of the difference in this condition, *BF_01_* = 4.93 (see ***Table 2*** for all BFs).

**Table 2:** Differences Between Switch Costs and Switch SOA (Diff Score) Separately for the Specific ITI/SOA increment Combinations.


	ITI 0 MS			ITI 250 MS			ITI 700 MS		

	SOA +20 MS	SOA +40 MS	SOA +60 MS	SOA +20 MS	SOA +40 MS	SOA –60 MS	SOA +20 MS	SOA –40 MS	SOA +60 MS

mean Diff Score	–39.78	39.84	76.76	10.10	50.20	94.19	24.78	70.30	86.74

t	–3.09*	3.53**	6.25**	1.04 ^n.s^.	4.28**	8.57**	3.69**	7.83**	5.64**

(dft)	(81)	(90)	(92)	(82)	(86)	(92)	(85)	(88)	(89)

BF_01_	1/10	<1/30	<1.100	4.93	<1100	<1/100	<1.30	<1/100	<1/100


*Note*: * *p* ≤ .05, ** *p* < .001. *BF*_01_ = the Bayes Factor in favor of the “null” Hypothesis, quantifies the evidence for the absences of the difference.

**Figure 4:**
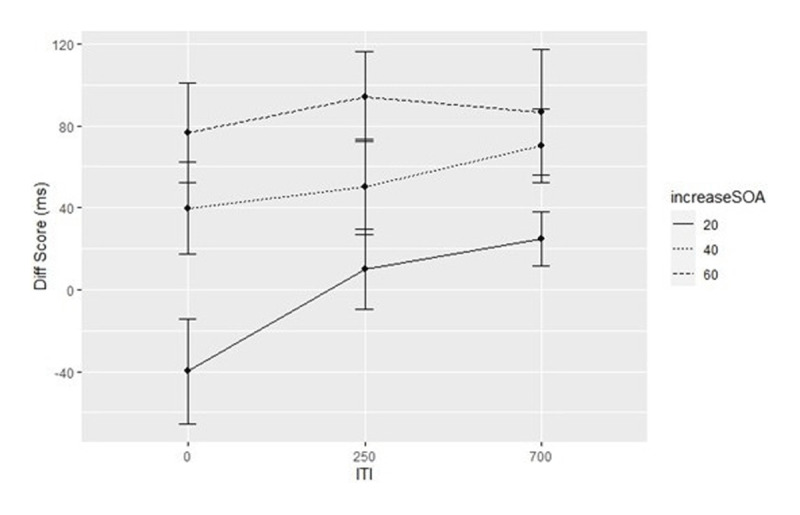
Means of Diff Score (median switch SOA – median RT switch cost) as a Function of ITI, and SOA increment. *Note*: Error bars represent a 95 % confidence interval for the mean.

A two-way repeated measures ANOVA was performed to evaluate the effects of ITI, and SOA increment on the Diff Score. There was a statistically significant two-way interaction between ITI and SOA increment, *F(4,260)* = 10.15, *p* < .001, *η_p_^2^* = .14, as is shown in ***Figure 4***. Also, the main effects for ITI and SOA increment were significant, *F(2,130)* = 9.08, *p* < .001, *η_p_^2^* = .12, and *F(2,112)* = 28.75, *p* < .001, *η_p_^2^* = .31, respectively.

The analysis of the difference between switch costs and switch SOA indicated the ITI 250/SOA +20 combination as the condition with the best trade-off indicated by the non-significant difference. Otherwise, our second indicator for the trade-off ability, that is the relation of the switch costs and switch rate on the individual level, revealed the strongest correlation between switch costs and switch rate in the ITI 250/SOA +60 combination, *r* = –.68, *p* < .001. However, the comparison of the correlation coefficients between all conditions, using the Fisher’s z transformation, revealed significant differences only between the correlation in the ITI 700/SOA +60 combination and correlations in the ITI 250/(SOA +20, +40) and ITI 700/SOA +40 combinations.^3^ The correlation of switch rate and switch costs in ITI 250/SOA +20 combination did not differ statistically significant from the strength of the correlations in other ITI 250/SOA +60 increment combinations (see Table A.2 in Appendix).

### REALTIONSHIP BETWEEN INDIVIDUAL’S TRADE-OFF ABILITY AND SWITCH RATE

**Figure 5:**
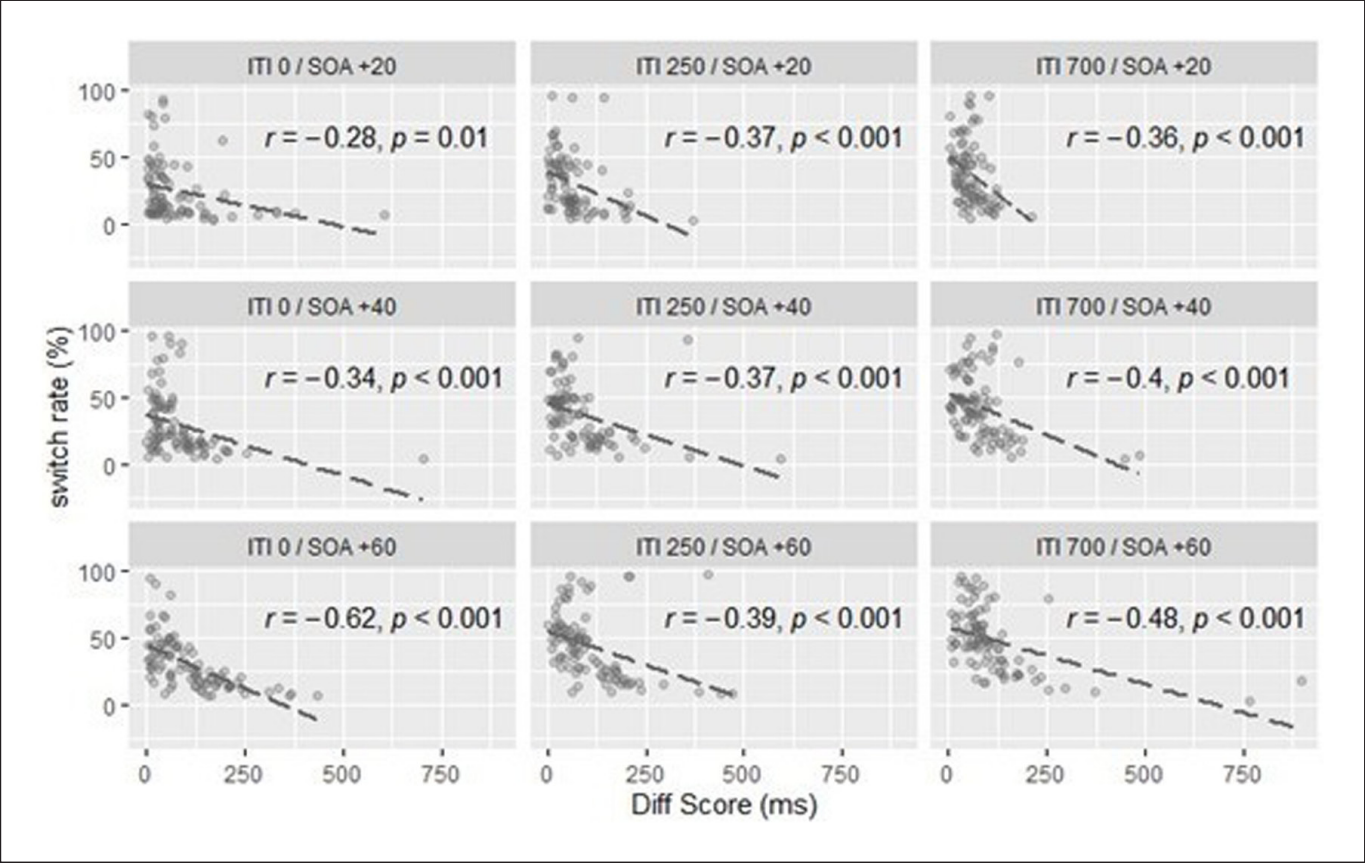
Relationship between individual trade-off ability (absolute value of Diff Score) and switch rate.

To determine the relationship between individual ability to balance costs and switching rates, we calculated Pearson product-moment correlation coefficients for each ITI/SOA increment combination. As illustrated in ***Figure 5*** Diff Score and switch rate correlated negatively across all ITI/SOA increment combinations. The correlation coefficients varied between *r* = –.28 and *r* = –.62, and all were significant, *p* ≤ .01.

## DISCUSSION

Previous self-organized task switching studies demonstrated that voluntary task selection is sensitive to both switch costs and the waiting time until a stimulus is presented in repetition trials (Mittelstädt, et al., 2018b, 2019). The observation that participants switched the task if the waiting time for a repetition stimulus corresponded to their switch costs supported the idea of strategic task selection that takes into account different costs. We explored this phenomenon in more detail in order to evaluate which SOA increment per repetition is best suited for which size of switch costs, so that participants can trade-off switch costs and waiting times most efficiently. Therefore, different ITIs (0 ms, 250 ms, 700 ms) and SOA increments (20 ms, 40 ms, 60 ms) were orthogonally manipulated. The present study revealed three main findings. First, we identified the ITI 250/SOA +20 combination as the condition that allowed the most precise tradeoff of switch costs and waiting time. Second, the correlation analysis showed that participants were able to adapt their task selection behavior to their task performance in all conditions. Third, greater individual ability to balance the cost efficiently is related to increased switch rate. We will address each point in turn.

The most efficient balancing of switch costs and waiting time was indicated by the absence of a significant difference between the average switch costs and the average switch SOA in the ITI 250/SOA +20 combination, whereas switch costs and switch SOA differed significantly for all other ITI/SOA increment combinations. This finding is consistent with the assumption that small SOA increments allow participants to match the waiting times more precisely to switch costs. Moreover, it seems that in this condition, participants used some kind of “local” strategy to balance switch costs and waiting times. When the switch costs match the switch SOA, we conjecture that participants trade-off the costs of switching and the cost of waiting for the repetition stimulus for the current trial on an equal basis – we therefore call the strategy a “local” strategy, because it only considers costs in one trial. In contrast, with the ITI 0/SOA +20 combination, we observed that participants switched tasks when the waiting time for repetition-related stimuli was slightly less than the switch costs. In other words in this condition, participants switched tasks before the waiting time for the repetition-related stimulus had increased to the extent of the switch costs. Possibly participants applied here a more “global” strategy by adapting their task selection behavior taking into account the fact that after each task switch, SOA increments start anew and consequently the subsequent task repetitions can be executed faster due to reduced waiting times. In contrast to a local strategy, we refer to this strategy as a more global strategy because waiting costs for several repetition trials may be taken into account.

It is obvious that the idea of two different efficiency strategies is still speculative. Nevertheless, it is an interesting observation that presumably two different strategies were applied under two different conditions, the local strategy under the conditions with the possibility to (partially) prepare the task switch in advance (i.e. ITI 250 and ITI 700), and the global strategy without this possibility (ITI 0). Given the evidence that self-organization of task sequence is cognitively demanding (Kiesel & Dignath, 2017), it might be possible that the global strategy is more advantageous in settings with larger switch costs. Whether the possibility of preparing the task switch influences the choice of the trade-off strategy remains an open question for future research.

Irrespective of the underlying strategical processes, the balancing of costs requires some kind of subjective awareness of switch costs and SOA changes. Bratzke and Bryce (2019) showed that participants were quite good at accurately introspecting about their switch costs. Moreover, using two different paradigms with externally controlled task switching, e.g. without the possibility of voluntary task selection, Bratzke and Bryce (2018) showed that participants were aware not only of their switch costs, but even of the decrease of the switch costs with increasing preparation time (ITI). Therefore it would be interesting to directly compare the introspection of switch costs and switch SOA in the voluntary task switching paradigm with the balancing of the respective costs.

Consistent with previous studies (Arrington & Logan, 2004; Arrington, 2008; Liefooghe et al., 2009; Mittelstädt et al., 2019), lower switch costs in conditions with long ITI were associated with the highest switch rates, supporting the assumption that the long ITI is used to prepare the task switch. On the other hand, the small difference (24 ms) between switch costs and switch SOA in the condition ITI 700/SOA +20 might indicate that although participants had the opportunity to prepare the task switch in advance, they decided to switch taking both costs into account. In other words, the task selection took place during the ongoing trial, considering switch costs and waiting time, and it is rather unlikely that task selection and preparation occurred before the trial started during the preceding ITI. Thus, we conjecture that our findings are more in line with the assumption of reduced switch costs due to passive decay of the repetition-related task set within the long ITI (Vandierendonck et al., 2010). And this passive decay facilitated the task switch in the subsequent trial where the actual task selection was based on the match between switch costs and switch SOA. On the other hand, it is also likely that task selection takes place partly before and partly during the trial. That is, the fact that in the ITI 700 conditions participants repeated the one task several times before switching to the other task indicates that preparation for the task switch starts during the ITI, but is not finished yet, so it continues into the ongoing trial. Of course such an assumption requires further testing, yet we conjecture it is likely that besides the strategic task selection, passive processes like the decay of the task set activation can also play a role in self-organized task switching.

The second finding—that switching behavior is correlated with switch costs—revealed that participants could flexibly adapting their task selection behavior to the factors influencing the task performance. More precisely, the correlation analyses between task performance and task selection for each ITI/SOA increment combination showed that participants differ in their switching behavior and that these differences correspond with their differences in switch costs. In detail, participants with higher switch costs switched tasks less frequently, and after longer waiting times, e.g. at larger switch SOAs. These observations are partly in line with the recent self-organized task switching studies. As in the results of Mittelstädt and colleagues (2018, 2019), switch costs correlated significantly negatively with switch rate, and we further extended the findings by demonstrating that switch costs correlated positively with switch SOA. Interestingly, Mittelstädt et al. (2018) already showed a tentative hint of the relationship between switch costs and switch SOA by using switch costs from the training blocks where the task switch was predetermined by the alternating-runs procedure in the correlation analysis. Furthermore, the flexible adaptation of task selection behavior to factors that influence task performance is also consistent with studies demonstrating that individuals flexibly adapt their task processing mode and response organization in multitasking to environmental requirements (for review see Fischer & Plessow, 2015; Lehle & Hübner, 2009) and to different task characteristics (Brüning and Manzey, 2018; Brüning, Reissland and Manzey, 2020) to maximize multitasking efficiency.

Yet, the correlational analyses were not helpful for determining which ITI/SOA increment combination allows for an optimal tradeoff of costs. Although the correlation coefficients were significant in all nine conditions, they were rather similar under all conditions. Thus, this measurement was unfortunately not useful for identifying the setting that allows participants to balance switch costs and switch SOA most efficiently.

Nonetheless, the positive correlation of switch rate with an individual’s ability to match time costs (i.e. switch costs and switch SOA) showed that individuals who are able to balance their costs more efficiently switch tasks more often. This finding supports the idea of strategic task selection and indicates that apart from specific characteristics of task or environment, the individual determinants may also have an impact on task switching performance. Comparably, investigations of inter-individual differences in multitasking have shown that individuals differ in the tendency to process tasks serially or simultaneously as well as to organize the task responses in a switching, blocking, or grouping manner (Reissland & Manzey, 2016, Brüning, & Manzey, 2018). Moreover, Brüning et al. (2020) demonstrated that the individual preference for the overlapping (i.e. simultaneous) task processing corresponds with a tendency to switch between tasks. Serial processing of the tasks, on the other hand, is related to the preference for the response blocking strategy. Importantly, application of the response organization strategy which did not correspond to the preferred task processing mode resulted in lower multitasking efficiency (Brüning et al., 2020). It seems that using an optimal strategy that meets either individual or environmental requirements improves multitasking efficiency. The present study did not investigate whether individuals with higher trade-off ability and higher number of voluntary task switches also performed task switching more efficiently, and this would be potentially fruitful avenue for further research.

Interestingly, the present results suggest that even in conditions with large switch costs the small SOA increments are sufficient to induce substantial switching behavior. Superficially, this finding seems to be inconsistent with previous research from Mittelstädt et al. (2019, Exp. 1a and 1b) in which they observed low switch rates (< 0.20 %) for large switch costs (301 ms and 224 ms, respectively). However, closer consideration suggests that the apparent contradictory results may be due to methodological differences. That is, in the study by Mittelstädt and colleagues (2019, Exp. 1a and 1b) participants performed the task in only one condition (ITI 0/SOA +50), whereas our blockwise ITI manipulation enabled participants to practice task switching under different conditions. It is conceivable that once task switching has been practiced in ITI 700 settings, it may become easier to switch in ITI 0 conditions leading to higher switch rates. This reasoning is in line with recent task-switching studies in which voluntary switch rates increased because participants were trained with task switches in forced-choice trials that were intermixed with free-choice trials (Fröber & Dreisbach, 2017).

In sum, the present research provided more parametric information about the scaling of a cost-benefit calculation in the self-organized task switching paradigm and showed how different costs fed into a common utility function that guides task selection. This refines previous findings specifying which ratio of waiting time and switch costs provides the most sensitive assessment of task selection. Thus, the self-organized task switching paradigm is a powerful instrument to investigate task selection and decision making in multitasking and can serve as a general purpose tool for understanding the relationship between task selection and task performance.

## DATA ACCESSIBILITY STATEMENT

Raw data of the reported experiments are available via the Open Science Framework: ***https://osf.io/7xqhc/***, doi: ***10.17605/OSF.IO/7XQHC***.

## ADDITIONAL FILES

The additional files for this article can be found as follows:

10.5334/joc.137.s1 Appendix Table A.1. Mean Switch Rate in each Free-Choice Block.

10.5334/joc.137.s2 Appendix Table A.2. Differences Between Z-transformed Correlation Coefficients for all ITI/SOA increment Combinations.

## Footnotes

We thank an anonymous reviewer for suggesting that we examine the relationship between individual ability to balance the time costs and individual task selection behavior.

As shown in ***Figure 3*** the correlation between switch costs and switch SOA in the ITI 700/SOA +60 combination was not significant, *r* = .18, *p* = .097. However, after the excluding the participant with extreme switch costs (975 ms) from the analysis, the correlation between switch costs and switch SOA reached significance, *r* = .45, *p* < .001, and the correlation between switch costs and switch rate became stronger, *r* = –.56, *p* < .001.

Using the outlier-corrected Pearson’s r in the ITI 700/SOA +60 combination for the comparisons revealed no significant differences between the correlation coefficients.
