# Supplementary material for: Scaling of the Parameters for Cost Balancing in Self-Organized Task Switching
Source: J Cogn. 2021 Jan 18;4(1):8. doi: 10.5334/joc.137 (PMC7824981; doi:10.5334/joc.137)
Supplement: Appendix Table A.1. — Mean Switch Rate in each Free-Choice Block. [file joc-4-1-137-s1.pdf]

Table A. 1

Mean Switch Rate in each Free-Choice Block.

Block number	***1***	***2***	***3***	***4***	***5***	***6***	***7***	***8***	***9***	***10***	***11***	***12***	***13***	***14***	***15***
Mean switch rate %	31	36	35	35	36	34	39	39	40	40	40	40	41	42	41
(SD)	(19)	(20)	(22)	(20)	(21)	(20)	(24)	(24)	(23)	(24)	(24)	(24)	(25)	(25)	(24)
