# Supplementary material for: Scaling of the Parameters for Cost Balancing in Self-Organized Task Switching
Source: J Cogn. 2021 Jan 18;4(1):8. doi: 10.5334/joc.137 (PMC7824981; doi:10.5334/joc.137)
Supplement: Appendix Table A.2. — Differences Between Z-transformed Correlation Coefficients for all ITI/SOA increment Combinations. [file joc-4-1-137-s2.pdf]

Table A. 2

Differences Between Z-transformed Correlation Coefficients for all ITI/SOA increment Combinations

	1	2	3	4	5	6	7	8	9
**1**	-	0.09	0.10	0.00	0.69	1.27	-0.09	0.91	-1.27
**2**	-	-	0.00	-0.10	0.62	1.21	-1.90	0.84	-1.40
**3**	-	-	-	-0.10	0.62	1.22	-0.19	0.85	-1.41
**4**	-	-	-	-	0.70	0.28	-0.09	0.92	-1.27
**5**	-	-	-	-	-	0.58	-0.80	0.22	-2.00*
**6**	-	-	-	-	-	-	-1.38	-0.36	-2.62*
**7**	-	-	-	-	-	-	-	1.02	-1.19
**8**	-	-	-	-	-	-	-	-	-2.23*
**9**	-	-	-	-	-	-	-	-	-

Note. 1= ITI 0/SOA +20, 2 = ITI 0/SOA +40, 3 = ITI 0/SOA +60, 4 = ITI 250/SOA +20, 5 = ITI 250/SOA +40, 6= ITI 250/SOA +60, 7 = ITI 700/SOA +20, 8 = ITI 700/SOA +40, 9 = ITI 700/SOA +60. Fisher's z transformation was applied. * $p \leq .05$, ** $p < .001$.
